# Navigation-assisted prone lateral transpsoas interbody fusion to potentially avoid approach-related complications

**DOI:** 10.3171/2026.4.FOCVID2651

**Published:** 2026-07-01

**Authors:** Camila A. Moreno Mesa, Steve S. Cho, Juan P. Giraldo, Juan S. Uribe

**Affiliations:** Department of Neurosurgery, Barrow Neurological Institute, St. Joseph’s Hospital and Medical Center, Phoenix, Arizona

**Keywords:** intraoperative navigation, lateral lumbar interbody fusion, minimally invasive spine surgery, neurovascular injury prevention, transpsoas approach

## Abstract

This video demonstrates CT-based navigated single-position lateral transpsoas interbody fusion with posterior percutaneous instrumentation. Intraoperative 3D navigation is integrated throughout the procedure to optimize trajectory planning, corridor docking, and implant placement. Navigated lateral surgery enables real-time visualization of the psoas and anterior vascular structures, helping mitigate neurovascular injury and anterior visceral complications during transpsoas access. By enhancing anatomical precision and reducing reliance on fluoroscopy, navigation may improve safety, streamline workflow, and further advance minimally invasive lateral fusion techniques.

The video can be found here: https://stream.cadmore.media/r10.3171/2026.4.FOCVID2651

## Transcript

In this video, we demonstrate a case of a prone lateral transpsoas interbody fusion at L4–5 and how navigation enhances safety during the lateral approach.

### 0:32 Clinical Presentation.

A 50-year-old male presented with axial low back pain and bilateral claudication exacerbated by standing and ambulation, partially relieved with sitting. He had failed conservative management, including physical therapy, anti-inflammatory medications, and injections. On physical exam, he had no sensory or motor focal deficit, with pain along the lower back and bilateral distal legs with a VAS-back of 6 and VAS-leg of 8.

### 1:00 Preoperative Images.

Preoperative MRI and CT demonstrated severe canal stenosis at L4–5 with bilateral foraminal stenosis and facet arthropathy. Flexion-extension radiographs demonstrated mild translational motion at L4–5 without gross instability.

### 1:17 Operative Plan.

Given the patient’s radiographic findings consistent with neurogenic claudication and mechanical back pain from L4–5 spondylosis, a single-position prone lateral transpsoas interbody fusion with navigational assistance was recommended. This approach would allow us to efficiently achieve indirect decompression and segmental stabilization at L4–5.^1^

### 1:38 Patient Positioning.

The patient is positioned prone on a Jackson table, with the abdomen freely suspended to minimize venous pressure and to maintain lumbar lordosis. Bolsters are used to allow coronal bending at the pelvis and chest to create a larger working window between the ribs and iliac crest. The hip and knees are flexed to elongate the psoas and move it posteriorly to place the lumbar plexus further posteriorly.^2^ Fluoroscopy is used to confirm the target level and define the disc level.

### 2:07 Surgical Procedure.

A dynamic reference array is secured to the posterior superior iliac spine, and intraoperative CT imaging is used to register the patient’s anatomy with the navigation platform. Posterior pedicle screws are placed percutaneously under navigational guidance. AP and lateral fluoroscopy confirm appropriate screw placement. Attention is then turned to the lateral approach. The abdominal musculature are dilated, and the retroperitoneal space is exposed and dissected. The psoas muscle is then palpated digitally to identify posterior bony landmarks, such as the transverse processes. The first dilator is advanced under real-time navigational guidance, which provides precise spatial orientation and allows visualization of key structures, including the vertebrae, disc space, psoas, and vascular structures.^3^ Because the navigation is based on postpositioning scans, the soft tissue structures can be accurately localized. Using navigation, the surgeon can dock anterior to the posterior one-third of the psoas, reducing the risk of encountering the lumbar plexus, which travel posteriorly along the psoas.^4^ Furthermore, navigation helps to prevent anterior trajectories, avoiding potential injuries to the vasculature and retroperitoneal structures. Neuromonitoring is still used as an adjunct safeguard. Triggered EMG provides functional confirmation that the lumbar plexus isn’t near the working corridor.^5^ Sequential dilation with triggered EMG is performed, and the retractor is placed. With the retractor in place, 3D navigation can be used again to confirm the extent of the psoas and delineate the safe working corridor. Fluoroscopy confirms docking within the recommended safe zone, corresponding to the middle half of the disc space at L4–5. This reduces lumbar plexus injuries while maintaining a safe distance from major vascular structures anteriorly.^6^ The discectomy and endplate preparation are then performed under navigational guidance, which helps to prevent off-trajectory insertion of working tools. Finally, the interbody graft is placed under navigation, and placement is confirmed under fluoroscopy. The remainder of the posterior work is completed, and standard closure is performed.

### 4:30 Postoperative Course.

Postoperatively, the patient endorsed significant improvements in the bilateral leg pain. He demonstrated minimal pain with hip flexion. He was discharged on postoperative day 1, and at follow-up visits, continued to endorse resolution of preoperative pain.

### 4:46 Conclusions.

In conclusion, the use of intraoperative navigation allows surgeons to accurately visualize the psoas and vasculature during prone lateral transpsoas lumbar interbody fusion to avoid neurovascular injuries. Along with the reduced reliance on fluoroscopy,^7^ the benefits of 3D navigation are manifold and should be considered in prone lateral surgeries.

## Acknowledgments

We thank the staff of Neuroscience Publications at Barrow Neurological Institute for assistance with manuscript and video preparation.

## Disclosures

J. Uribe reported royalties from NuVasive and SI-BONE; and consulting fees from SI-BONE, Misonix, Viseon, and Mainstay Medical.

## Author Contributions

Primary surgeon: Uribe. Assistant surgeon: Cho. Editing and drafting the video and abstract: Moreno Mesa, Cho, Giraldo. Critically revising the work: all authors. Reviewed submitted version of the work: Uribe. Approved the final version of the work on behalf of all authors: Uribe. Supervision: Uribe.

## Supplemental Information

### Patient Informed Consent

The necessary patient informed consent was obtained in this study.
